# Imaging Technologies for Cerebral Pharmacokinetic Studies: Progress and Perspectives

**DOI:** 10.3390/biomedicines10102447

**Published:** 2022-09-30

**Authors:** Weikang Ban, Yuyang You, Zhihong Yang

**Affiliations:** 1 Institute of Medicinal Plant Development, Chinese Academy of Medical Sciences and Peking Union Medical College, Beijing 100193, China; 2 Beijing Institute of Technology, School of Automation, Beijing 100081, China

**Keywords:** cerebral pharmacokinetics, imaging technologies, optical imaging, radiographic autoradiography, radionuclide imaging, mass spectrometry imaging

## Abstract

Pharmacokinetic assessment of drug disposition processes in vivo is critical in predicting pharmacodynamics and toxicology to reduce the risk of inappropriate drug development. The blood–brain barrier (BBB), a special physiological structure in brain tissue, hinders the entry of targeted drugs into the central nervous system (CNS), making the drug concentrations in target tissue correlate poorly with the blood drug concentrations. Additionally, once non-CNS drugs act directly on the fragile and important brain tissue, they may produce extra-therapeutic effects that may impair CNS function. Thus, an intracerebral pharmacokinetic study was developed to reflect the disposition and course of action of drugs following intracerebral absorption. Through an increasing understanding of the fine structure in the brain and the rapid development of analytical techniques, cerebral pharmacokinetic techniques have developed into non-invasive imaging techniques. Through non-invasive imaging techniques, molecules can be tracked and visualized in the entire BBB, visualizing how they enter the BBB, allowing quantitative tools to be combined with the imaging system to derive reliable pharmacokinetic profiles. The advent of imaging-based pharmacokinetic techniques in the brain has made the field of intracerebral pharmacokinetics more complete and reliable, paving the way for elucidating the dynamics of drug action in the brain and predicting its course. The paper reviews the development and application of imaging technologies for cerebral pharmacokinetic study, represented by optical imaging, radiographic autoradiography, radionuclide imaging and mass spectrometry imaging, and objectively evaluates the advantages and limitations of these methods for predicting the pharmacodynamic and toxic effects of drugs in brain tissues.

## 1. Introduction

Pharmacokinetics is the quantitative study of the dynamics of absorption, distribution, metabolism and excretion (ADME) of drugs in living organisms, as well as their dose–time relationships. The efficacy of central nervous system (CNS) drugs depends on the extent to which they enter the brain tissue, i.e., the concentration of drugs in the brain. The pharmacokinetic process of CNS drugs is clinically monitored to find the optimal dose, so that the patient’s blood concentration is within the therapeutic window, thus, indirectly controlling the drug concentration in the brain, the target organ. Physiologically, the blood–brain barrier (BBB), a special structure of brain tissue, restricts the entry of harmful substances, whereas the tightly packed cells and multiple uptake or efflux transporters on the barrier also limit the distribution of drugs in the brain. BBB has become a major obstacle to intracerebral drug therapy, with approximately 98% of small-molecule drugs and nearly 100% of large-molecule drugs not passing through [1]. When the permeability of BBB to drugs is altered, the blood concentration obtained from therapeutic drug monitoring may not correctly reflect the drug concentration in the brain, posing a potential hazard to the safety and efficacy of drug therapy [2]. A study by Dagan et al. [3] suggests that simply using blood levels as a surrogate marker for drug concentrations in the CNS can lead to underdosing. Additionally, brain tissue, as the major part of the CNS in animals, is also a fragile organ due to the lack of energy reserves. When non- CNS drugs enter brain tissue and produce off-target effects, they are likely to damage the higher level of neural activity in the animal. Therefore, studying the metabolic kinetics of drugs in brain tissue can determine whether CNS drugs reach the brain or finer structures and if they reach safe and effective concentrations in the brain, as well as the disposition of non-CNS drugs in the brain, which is of great significance in the pharmacodynamic and toxicological prediction of drugs in the brain and plays an important role in reducing the risk of new drug development.

**Figure 1:**
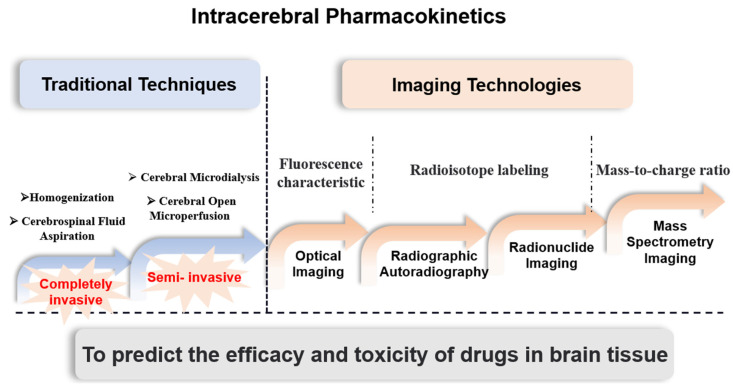
Development of intracerebral pharmacokinetic strategies. This review focuses on the development trends and recent advances of some imaging-based cerebral pharmacokinetic study methods, objectively assesses the advantages and limitations of various technical methodologies, and provides a reference for predicting the pharmacodynamic and toxic effects of drugs in brain tissue.

As knowledge of the fine structure of brain tissue has advanced over the decades, brain pharmacokinetic studies have become more sophisticated and improved (Figure 1). Most traditional intracerebral pharmacokinetics techniques, such as brain tissue homogenization and cerebrospinal fluid aspiration, are completely invasive and the unexplained lack of stability in data and spatial resolution have become major difficulties. Cerebral microdialysis (CMD), which emerged and was developed in 1974 and cerebral open microperfusion (cOFM), which was refined in this paradigm, both represent semi-invasive methods that enable continuous in vivo sampling and have a limited impact on the organism [4]. As a result of the provision of free drug collection, CMD has become an integral part of pharmacokinetic studies of neuroactive drugs.

However, the impact of the invasion of these semi-invasive techniques on the integrity of the BBB may lead to bias in the quantification of drug concentrations in the brain, thus, affecting the experimental results, which led to the birth of non-invasive technical methods. In addition to improving spatial resolution, non-invasive imaging techniques have provided the ability to capture the dynamic processes of drugs in various fine structures within the brain as well. Optical imaging techniques, which exploit the fluorescence characteristics of drug molecules for tracking and imaging, have gradually changed from ex vivo tissue imaging to in vivo fluorescence imaging and have undergone further development. Depending on the type of radiograph, radiographic autoradiography and nuclear imaging can be used to study pharmacokinetics in the brain and the latter is capable of noninvasive imaging in vivo and is now widely used in clinical treatment. Nuclear imaging techniques, such as positron emission tomography (PET) and computed tomography (CT) or magnetic resonance imaging (MRI), are commonly used to visualize drug distribution and therapeutic efficacy. While the above imaging methods are effective for the application for which they are intended, they fall short of the potential of more recent mass spectrometry imaging (MSI) techniques. The cumbersome and time-consuming radiolabeling process and lack of imaging resolution and specificity have become major obstacles to its penetration into pharmacokinetic studies in the brain. MSI has the unique advantage of the mass-to-charge ratio of mass spectrometry, which enhances the specificity of imaging and allows simultaneous analysis and localization of multiple molecules. Further than qualitative and quantitative assessment, MSI is able to correlate with function and co-localize drugs with endogenous molecules to provide references for disease progression, therapeutic efficacy and medication safety.

## 2. Optical Imaging for Assessing Intracerebral Pharmacokinetics

In the mid-20th century, weber used the fluorescence properties of molecules, as well as absorption and emission spectra, to pinpoint molecular dynamics and reveal the kinetic parameters of biologically relevant processes, such as enzyme binding. These experiments laid the foundation for the quantitative application of fluorescence spectroscopy in biochemistry. By the end of the 20th century, many small fluorescent molecules were discovered and classified to be repurposed as markers for binding to other molecules. The discovery and application of fluorescent molecules has led to the natural application of fluorescent molecules with visual binding in tissues for numerous biodistribution studies. Not least of these is visualization in the CNS, i.e., brain tissue. In recent decades, theoretical, instrumental and technological developments have made it possible and widespread to visualize drugs and their metabolites in brain tissue.

**Figure 2:**
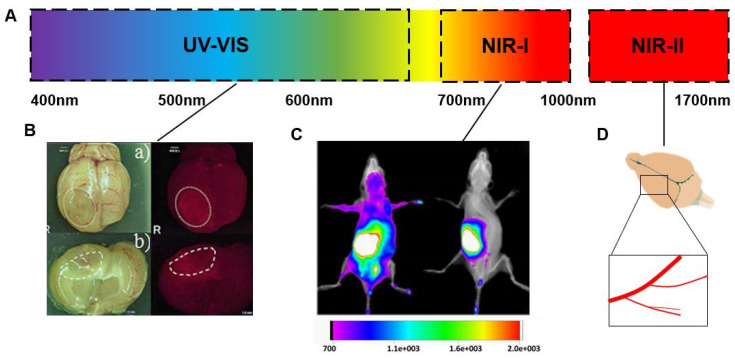
Optical imaging of brain tissue in visible light, NIR-I and NIR-II wavelengths. (**A**) Schematic diagram of visible-light, NIR-I and NIR-II wavelength intervals. (**B**) Macroscopic view of (a) whole brain and (b) coronal images after administration of 5-aminolevulinic acid (5-ALA) in a rat vasogenic edema model, as well as fluorescence images obtained using a 610 nm long path filter. Reprinted with permission from Ref. [6]. © 2009, TakaoTsurubuchi. (**C**) The in vivo biodistribution assay of NANG/PLGA/DTX/ICG NPs was analyzed in terms of fluorescence intensity (the fluorescence intensity scale below ranges from cold to warm colors indicating low to high intensity, and e +003 denotes 10 to the third power). Extensive retention of targeted nanoparticles in brain regions was observed. Reprinted/adapted with permission from Ref. [18]. © 2015, Yongwei Hao. (**D**) NIR-II fluorescence through skull microscopic imaging using a 1100 nm long path filter was used to monitor cerebral vascular changes in the same region of the mouse brain before and after the MCAO model to verify model success [24].

Optical imaging uses the ability to excite photons from the UV to IR spectrum generated by bioluminescence or fluorescence to become a powerful molecular imaging modality in disease diagnosis and treatment [5]. Depending on the spatial resolution and tissue penetration of the optical imaging system, different imaging modes can be used for the analysis of ex vivo and in vivo brain tissue (Figure 2A).

### 2.1. Ex Vivo Imaging of the Brain

Fluorescence microscopy allowed for qualitative observation of the distribution of fluorescence-containing substances in the brain. This method could observe the distribution of the photosensitizer 5-ALA in the brain of the rat model of vasogenic edema. The strongest red fluorescence was detected in the edematous areas, as well as in the white matter of the ipsilateral corpus callosum 6 h after injection, which can be quantified when combined with the detection of fluorescence brain tissue homogenates (Figure 2B) [6]. Fluorescence imaging of isolated brain tissue can also measure the ability of different nano-agents to penetrate the blood–brain barrier by quantifying the fluorescence intensity of the brain [7]. The fluorescence intensity of the fluorescently Ce6-labeled bCDsu-thioketal-memantine (bCDsuMema) nanoparticles was strongest in the brain, suggesting that nanoparticles can effectively target the brain, providing a promising candidate for the inhibition of Alzheimer’s disease [8]. Alata et al. [9] used in situ brain perfusion (ISPB) combined with optical brain imaging and distribution volume evaluation to determine brain accumulation of sdAbs fused to mouse Fc (sdAb-mFc) after penetration disruption of BBB. The BBB-opened right hemisphere showed a significant increase in total fluorescence intensity and volume of distribution in the isolated brain compared with buffer-only perfusion of A20.1-mFc, which was 30-times and 6-times higher than buffer-only perfusion, respectively. Furthermore, detection of monoclonal antibodies in the brain microvasculature and brain parenchyma showed that approximately 25% was segmented into the brain parenchyma and 75% was segmented into the brain vasculature. Multiple assays were fused to obtain a comprehensive characterization of antibody brain tissue uptake and distribution.

In addition to qualitative observation of whole brain tissue, fluorescence microscopy of frozen brain tissue sections is also more common to provide a reference for drug localization of finer structures. Israel et al. [10] studied the pharmacokinetics and biodistribution of the nanoconjugate P/LLL/AP2/rh in different regions of the brain and at multiple time points by optical images combined with immunofluorescence staining. To ensure that P/LLL/AP2/rh enters the brain parenchyma through BBB, a fluorescence intensity analysis was performed in regions that do not contain the vascular system (lectin labeling). Relative fluorescence intensity values of the perivascular parenchyma of the cerebral sagittal sinus in the cerebral cortex were analyzed 30–480 min after drug injection to determine drug accumulation and clearance and the results showed a different time dependence than serum pharmacokinetics. Fluorescence in brain tissue had a high onset time and remained quasi-stable for 120 min, with clearance occurring after 240–480 min. Fluorescence intensity can be quantified in selected slice regions, such as brain regions associated with disease, somatosensory cortex Ⅱ/Ⅲ, midbrain thalamus and hippocampal CA1-3 cell layer.

Researchers are now able to insert the luciferase gene into cells of interest and apply it to animal models to immediately identify the organs in which they reproduce, as well as any changes in biodistribution that may occur over time. Light is released in the interaction between the protein produced by the engineered cell and the given substrate. Taylor [11] established a bioluminescence imaging strategy with a large stokes-shift monomeric orange fluorescent protein (LSSmOrange) as a receptor and NLuc as an energy (light) donor to the fluorescent acceptor. The LSSmOrange/NLuc system combined with firefly luciferase was used to monitor the biodistribution of multiple cell populations in vivo. The left cardiac ventricle was injected with a mixture of MSCs expressing LSSmO_NLuc and RAWs expressing FLuc to capture changes in signal intensity. Raws proliferated significantly and earlier in the head, between day 2 and day 15 after furimazine administration. However, it was possible that the cells revealed by these images were trapped in the brain capillaries and had not yet reached the brain parenchyma.

### 2.2. In Vivo Imaging of the Brain

Exploring the biodistribution of drugs within tissues has traditionally relied on postmortem tissue imaging and tissue homogenization for quantification but has also seen the development of methodologies that do not require tissue excision. In vivo imaging helps identify suitable therapeutic targets and assess biodistribution and PK, as well as on-target and off-target effects. Therefore, in vivo imaging has become an integral and important component of drug discovery, development and clinical evaluation for CNS diseases.

Early researchers applied fluorescence imaging systems to enable exogenous administration of drugs to reflect changes in endogenous substances. For example, based on fluorescence lifetime contrast unmixing, the exogenous compound dihydroethidium (DHE) [12] was used as a fluorescent probe to monitor in real time the superoxide production capacity of specific regions of living brain tissue, including the cerebellum, hippocampus and cortex [13]. Recently, Zhou et al. [14] used the lumina IVIS Ⅲ imaging system to focus on the brain targeting ability of the nanoparticle Gal-NP@ Cy5-siRNA, demonstrating its rapid uptake and distribution processes in the form of continuous images. In vivo brain tissue imaging increases the inherent attenuation of skin and skull imaging compared to ex vivo brain tissue imaging and recent new waveband imaging focuses on addressing this issue.

#### 2.2.1. NIR-I Imaging

Fluorescence imaging uses the ability of light from the ultraviolet to infrared spectrum to excite molecules, which, when activated, emit longer-wavelength photons that can be easily detected by specialized sensors. However, when UV or visible light penetrates the tissue layers, the light is easily and strongly absorbed or dispersed by the pigments in the tissue (e.g., melanin, hemoglobin), hindering image capture and analysis. In this case, the porphyrin ring of heme is particularly important due to its extremely strong absorption band and a weaker absorption peak region around 400 nm and 550 nm, respectively [15]. These interferences are more pronounced in the visible spectrum (400 nm–700 nm) than in the near-infrared (700 nm–2000 nm) spectrum; therefore, the near-infrared region is considered an optical or therapeutic window [16]. Spectral imaging in the near-infrared (NIR) range (700–1000 nm) maximizes tissue penetration and minimizes self-fluorescence from non-target tissues compared to conventional visible imaging [17] and is widely used for preclinical in vivo imaging studies and has become the modality of choice for in vivo studies.

Using in vivo NIR imaging, NANG/PLGA/DTX/ICG NPs modified with the brain-targeting peptide angiopep-2 demonstrated the “uptake—distribution—clearance” process in the brain region, with the drug concentration in the brain low at 1 h, peaking at 2 h and decreasing again at 4 h. Brain distribution imaging provides a basis for the application of brain-targeting nanoparticles in glioma [18] (Figure 2C). Ronald et al. [19] monitored the biodistribution kinetics of the near-infrared monoamine oxidase inhibitor (NMI) based on total tissue signal intensity at different time points. The NMI imaging signal of intracranially implanted glioma mice GL26 showed that the maximum signal was reached at 48 h in the brain, tumor and other important organ tissues, with the best brain imaging signal (tumor/non-tumor ratio > 3.5) and small off-target distribution. The results not only illustrate the intra-brain and intra-tumor distribution in the treatment and diagnostic monitoring of NMI glioma but also provide a reference for potential off-target effector organs of NMI in future studies. In clinical practice, it is often difficult for surgeons to distinguish tumors from normal brain tissue due to their infiltrative nature and similar appearance. New strategies have been applied to access the tumor through the blood–brain barrier and the blood–tumor barrier (BTB), using contrast agents intraoperatively to help outline the tumor margins for complete and precise resection. One strategy includes the use of the peptide/fluorophore adjuvant Tozuleristide, which is a tumor-specific NIR-targeting agent that has entered human clinical trials [20]. Capable of selective binding to glioblastoma tumor tissue in the NIR range, it has low autofluorescence and good tissue penetration in the NIR range.

#### 2.2.2. NIR-II Imaging

In the last decade, developments in near-infrared imaging have focused on the so-called first biological window, NIR-I (700–1000 nm). Simulation and modeling studies in 2003 for optical imaging in turbid media, such as tissue or blood, showed that it is possible to improve the signal-to-noise ratio by more than 100-fold using quantum dot fluorophores with emitted light of 1320 nm [21]. However, until recently, the development of biocompatible fluorescent probes allowed the technique to be further explored in the second biological window (NIR-II), also known as short-wave infrared (SWIR, 1000–1700 nm) [22]. Its significant improvement in tissue penetration and image resolution of biological samples has high potential for clinical translation [23].

In recent years, the development and application of SWIR imaging systems in the CNS field have enabled the visualization of cerebral microvessels (less than 10 μm in diameter) that pass through the intact skull of mice [24] (Figure 2D) and the main vessels of the rat brain [25]. There have also been advances in various fluorescent emitters used for this process, such as organic fluorescent dyes, SWCNTs [26] and quantum dots (QDs) [27]. Due to the low light scattering of the NIR-II fluorescence signal, the biological tissue itself has less NIR-II fluorescence, it has deeper penetration depth, higher spatial resolution and better signal-to-background ratio (SBR). After intramuscular injection of ICG, NIR-II was able to penetrate 300 μm depth and identify tiny capillaries as small as 2.66 μm in diameter and image SBR can reach 4.48 [24]. Organic nano-fluorophores (named p-FE) emit a bright 1100 nm NIR-II fluorescence for noninvasive in vivo tracking of circulating blood flow in mouse cerebral microvasculature, with imaging depths up to 1.3 mm [28]. Based on anatomical visualization and blood flow analysis of brain tissue, SWIR imaging can be used to assess vascular pathology following cerebrovascular tumors or cerebrovascular injury. Starlike polymer brush-based ultrasmallTQFP-10 NPs with bright NIR-II fluorescence for efficient in situ glioblastoma (GBM) imaging by prolonging blood circulation and enhancing tumor accumulation [29]. The above applications are based on the visualization of the distribution of molecules in the cerebral vasculature and tumors in the brain, and visualization of the distribution of fluorescent molecules in the NIR-II region in the brain helps to identify suitable therapeutic targets for CNS. The field is evolving rapidly and new frontier imaging at wavelengths beyond 1700 nm (between 1750 and 2500 nm) is already being addressed and further improvements in tissue transparency are expected.

## 3. Autoradiography for Assessing Intracerebral Pharmacokinetics

Radiographic autoradiography is a technique for visualizing radiolabeled molecules or molecular fragments using X-ray films, fluorescent imaging plates, beta imaging systems or nuclear photoreceptor emulsions and has been used for the past several decades for the localization and quantification of drugs in tissues and cells. Data acquired by this technique form the basis for many safety assessments, such as whether drug molecules reach the target organ, show potential for off-target accumulation and toxicity or have a specific affinity or bind to specific tissues. For these purposes, ex vivo in situ imaging modalities, such as whole-body radiographic autoradiography and microradiographic autoradiography, are used to provide the detailed tissue distribution and subcellular tissue localization information needed for drug discovery and development.

### 3.1. Whole-Body Autoradiography (WBA)

Whole-body autoradiography (WBA) is an accepted technique for obtaining information on the spatial distribution of compounds in animals by radiolabeling the drug and, thus, visualizing it in animal tissue sections to obtain information on the distribution of the drug in the organ of interest. Quantitative whole-body autoradiography (QWBA), which combines WBA with phosphor screen technology, provides visualized spatial-distribution images of radiopharmaceuticals and matched radiopharmaceutical tissue concentration and has become an important tool for qualitative and quantitative characterization in pharmacokinetic studies.

QWBA is now well established and has become very common and is the most important method for assessing tissue distribution in nonclinical studies for drug development [30]. QWBA provides high-resolution images of the spatial distribution of drug-related radioactivity in experimental animals and matches highly reproducible quantitative tissue concentration data in areas as fine as 50–100 μm. Kertesz [31] compared the propranolol content of brain tissue from WBA sections by quantitative phosphor screen analysis with radioactive DESI-MS/MS signals and showed that both quantitative techniques analyzed the same amount of propranolol in brain tissue. The administration of radiolabeled drugs followed by execution at specific time points and rapid freezing to obtain in situ samples of all organs, tissues, body fluids, chyme and excreta has also become a major advantage of QWBA. QWBA can provide true tissue level in situ concentrations, with minimal sample handling/alteration for the investigator’s purpose of probing the true condition of the tissue. Pan et al. [32] observed the highest distribution of ^14^C-methamphetamine (^14^C-METH) in mouse brain tissue at 5 min of intravenous administration (specific activity of 52.41 ± 4.5 nCi/g) in whole radiographic autoradiographic sections, followed by rapid decrease, and the elimination rate slowed down from 15 min onwards. The overall radiographic autoradiographic sections were only used to visualize and quantify the radiopharmaceutical content in the sagittal plane of brain tissue, while the examination of the coronal plane and fine structure of brain tissue is still insufficient. Pan et al. followed up by coronal sectioning of the mouse head to examine the concentration of radiopharmaceuticals in different coronal sections of the same brain tissue to determine the distribution density of ^14^C-METH in different areas of brain tissue and obtained the conclusion that METH rapidly entered the gray matter of the brain with blood flow and was widely and uniformly distributed.

To focus on drug distribution and course of action in specific regions of brain tissue, researchers can also rapidly dissect brain tissue after animal execution for subsequent freezing and imaging operations. As early as 1990, David [33] performed a quantitative radiographic autoradiographic analysis to investigate the distribution of 5-HT_3_ binding sites of [^3^H]quipazine in brain tissue by combining coronal and sagittal sections of brain tissue. Radiographic autoradiography was compared with Nissl staining and rat brain localization atlas and 29 binding sites were identified in rat brain tissue, among which the most obvious and dense 5- HT_3_-specific binding regions in the medulla were the nucleus tractus solitarius and the caudal part of the trigeminal nerve bundle. In continuation of the radiographic autoradiography of isolated brain tissue, Faresjo [34] examined the more complete brain pharmacokinetics of the bispecific TfR1-conjugated antibody in sagittal brain sections in 24 h. The data showed a stable intracerebral distribution of [^125^I]mAb3D6-scFv8D3 (first eight hours after injection), with a maximum intracerebral concentration at 8 h. This was followed by a net elimination phase.

However, the lack of specificity of WBA is common to all technical approaches that rely on radiolabeled compounds, which provide data only on total radioactivity and not specifically on the parent compound. In other words, the concentration of radioactivity is not always equivalent to that of the original compound being labeled and it may also include radioactivity associated with metabolites and/or degradation products. Kertesz used HPLC and radiochemical assays to determine the relative levels of propranolol and metabolites based on WBA sections [31]. The small-molecule protein NBN is a member of the family of glial cell-derived neurotrophic factor and the distribution of total ^35^S-NBN radioactivity in the brain was extremely low (less than 0.3% of the initial dose), with an increase in radioactivity of brain tissue at the time point after 5 min, most likely due to the recycling of radiolabeled methionine and cysteine to other proteins [35]. In addition to this, signal recognition by WBA requires high doses of the drug molecule, so high specificity–low-dose binding sites are often not recognized and high specificity–low-dose sites are often found in brain tissue, spinal cord and pituitary gland [36]. Baumann [37], when determining the distribution of [prop-^14^C] BAY 1025662 in the rat brain, found that brain exposure was extremely low, with brain tissue radioactivity concentrations only slightly above the detection limit on day 7 after administration and the C_max_ and AUC of brain radioactivity were approximately 1.4% of the corresponding parameters in plasma, presumably as a result of residual blood in the cerebral vasculature rather than infiltration into brain tissue.

### 3.2. Microautoradiography (MARG)

Microautoradiography (MARG) is another radiographic autoradiography technique that was first designed to improve the resolution obtained with radiographic autoradiography. After early insights and refinements by Belanger [38] and Pelc [39] on its application to tissue preparation and nuclear emulsion, MARG enabled the localization of radiolabeled compounds to even smaller tissues or cells. Wang et al. [40] applied this method to the observation of intra-tumor drug uptake in F98 glioma-bearing rats, using the intensity of radioactivity of ^18^F-FBPA-Fr in tumor and normal brain tissue as a quantitative index of its uptake. The tumor–normal brain tissue radioactivity intensity ratio reached a maximum (3.45) at 0.5 h after injection and slowly decreased over time, confirming that the uptake of ^18^F-FBPA-Fr by tumor tissue was higher than that of surrounding normal brain tissue, raising expectations for clinical boron neutron capture therapy with BPA-Fr.

A new application of in vitro radioautography for the quantitative determination of drug concentrations is in the field of tissue receptor studies. MARG excels in providing in situ localization of the radioactivity of receptor-bound drugs in various cell types, which has predictive value for specific drug targeting. It has been widely used to provide important information about the cellular mechanisms of drug distribution and localization of receptor binding. In terms of manipulation, it is similar to whole-body radioautography, except that its thicker frozen sections of tissue (4–10 μm) are mounted onto slides pre-coated with nuclear emulsion for imaging and the slides are subsequently stained using traditional histological stains, such as immunohistochemical (IHC) staining techniques, to provide positive co-localization of drug-derived radioactivity to known cell types and/or receptor markers. Microradiographic autoradiography was able to localize the bound cell types with a radioactive signal (presented as silver grains). Mizoguchi et al. [41] used this method in their 2014 study of the specific binding characteristics of 18β-glycyrrhetinic acid (GA) in the rat brain. Through quantitative radioautography analysis of several regions of the brain, the degree of specific binding in different regions of the brain was classified into three levels according to the radioactive density, which were higher, moderate and lower density. These three classes correspond to the hippocampal region; the caudate, vomeronasal, amygdala, olfactory bulb, cerebral cortex, thalamus and midbrain regions; and the brainstem and cerebellar regions. MARG showed that [^3^H]GA signals in the hippocampus were distributed in small non-neuronal cells similar to astrocytes and the merging of MARG images with microscopic images of immunohistochemical images of glial fibrillary acidic protein (GFAP) showed evidence of specific binding sites in specific regions of the brain, suggesting that astrocytes are the primary target cells of GA. These findings have important implications for understanding the pharmacological effects of GA in the brain after glycyrrhiza entry and exploration of its cellular mechanisms. In addition to cell type, binding to receptors in the CNS can also be localized with the help of this method. Farar et al. [42] examined the binding of D1-like dopamine receptors (DR), toxicomycin receptors (MR) and the total number of receptors in the CNS of rats exposed to the addictive drug methamphetamine (MA), focusing on intracerebral structures associated with drug addiction and cognition, such as the mesolimbic system and hippocampus.

The localization of compounds in cells and tissues demonstrated by MARG can provide a strong set of evidence for precise drug distribution and cellular mechanisms, but the processing time to obtain results from MARG is difficult to measure. This is because each tissue must be processed and evaluated differently depending on the level of radioactivity present. Exposure times can vary from days to weeks or even months for optimal results, a time course that may be impractical for the evaluation of multiple samples. In addition to this, control of artifacts during the imaging process is essential and any problem that arises can undo months of work.

## 4. Radionuclide Imaging for Assessing Intracerebral Pharmacokinetics

X-ray computed tomography, also called computed tomography (CT scan), is a medical imaging technique that uses radiation to obtain a view of the inside of the body, using tomography generated by computer processing for medical imaging. In 1973, X-ray computed tomography [43] was introduced to facilitate the development of the field of nuclear imaging techniques, particularly positron emission tomography (PET) and magnetic resonance imaging (MRI), and their introduction provided researchers with an unprecedented opportunity to study the neurobiological correlates of the behavior of living organisms [44] and also provided a visualization scheme of the pharmacokinetics in the brain.

### 4.1. Positron Emission Tomography (PET)

Positron emission tomography (PET) has become a powerful imaging modality since its introduction in the late 1970s, with the ability to detect molecular tracers with high sensitivity. Compared to WBA, in vivo noninvasive nuclear imaging provides realistic in vivo drug visualization information and real-time drug distribution tracing, providing in vivo quantitative spatially resolved pharmacokinetic and pharmacodynamic measurements for a wide range of molecular drugs in the brain [45].

PET is assayed for quantification based on decay of the radioactive material activity. Brain uptake of radioligands is described by the area under the time–radioactivity curve (AUC), which can be used to assess transporter protein-mediated drug–drug interactions (DDI). One study [46] evaluated the effect of cyclosporine a (CsA) on the kinetics of [^11^C]-dLop in the brain of baboons. Inhibition of P-gp by injection of CsA resulted in a 12-fold increase in AUC_10–30_ from baseline values and a significant increase in brain tissue radioactivity, with a gradual increase in radioactivity over time, even after the distribution period. Small-animal PET has a positional resolution of 1–2 mm and can detect drug concentrations in different regions in defined organs/tissues. PET can be used to observe the uptake of radiotracers in brain tumor tissue and _L_-l-[^18^F] FETrp penetrates the BBB in a mouse model of glioma and accumulates in 73C glioma (4.1 ± 0.7% ID/g) [47]. The uptake of radiotracer by gliomas is significantly higher than that of normal brain tissue, allowing for precise localization of brain tumors and strongly supporting the clinical transformation of brain tumors.

Since PET does not provide chemical resolution, the same radioactive signal in vivo makes it difficult to distinguish between the parent drug and its radioactive metabolites. To quantitatively characterize PET data, constant conversion of radiopharmaceuticals between plasma and different tissues requires concentration–time profiles of unmetabolized labeled drugs in arterial plasma (i.e., arterial input function). This function provides the linkage and relationship between brain–blood concentrations of radioactive substances. As determined by the plasma input function and time–activity curve, the ratio of brain to plasma concentration at equilibrium is the total distribution volume. Brain–plasma concentration ratios affect the safety and efficacy of drug therapy. PET can provide useful assistance; for example, the application of PET in vivo imaging is necessary for clinical human drug brain–blood concentration monitoring [48]. However, continuous arterial blood sampling is difficult for the operator and has a great impact on the physiology of experimental animals. Moreover, due to the short half-life of some radionuclides, it is not accurate to detect constant concentrations of the parent drug late in the scan. Therefore, the arterial input function is also the most error-prone aspect of PET experiments. To avoid the effects of arterial blood sampling on PET, alternative methods, such as vena cava [49,50], microfluidic blood sampling [51], arteriovenous shunts combined with γ-counters [52] and β-probe insertion into the femoral artery [53], have been investigated to complete the input. After appropriate biomathematical correction of the arterial input function, the total concentration of radiolabeled drugs reaching the brain tissue can be measured directly. However, the nonspecific binding component of highly protein-bound compounds is not excluded. An experiment [54] combined PET imaging with in vitro equilibrium dialysis to determine the nonspecific binding components of unlabeled drugs in plasma and tissues to estimate the free brain concentration of drugs and assess their transport processes. [^11^C]-Loperamide itself lacks brain penetration and shows significantly higher uptake in the presence of the known P-gp inhibitor CsA. On the contrary, the structural pituitary itself, present outside the blood–brain barrier, has high uptake and is not affected by P-gp regulation. This combined means analysis excludes nonspecific binding components and may become a useful tool for CNS drug development. In addition to this, investigators have attempted to separate protein-free and bound parental tracers and their radioactive metabolites by developing new tracers to obtain the correct output values [55].

### 4.2. Magnetic Resonance Imaging (MRI)

Magnetic resonance imaging (MRI) is a versatile imaging tool with high soft tissue contrast that noninvasively visualizes details of internal structures located at greater depths with high resolution and can be used as a method to monitor the pharmacokinetics of drug entry processes into the brain. Strong magnetic field gradients cause nuclei at different locations in the organism to rotate at different speeds, thus, providing three-dimensional spatial information. MRI provides excellent contrast between different soft tissues of the body, which makes it distinctive for imaging the brain, muscles, heart and cancer.

MRI is used mainly to examine the distribution of the molecule in the brain. Valdez [56] determined the volume of the brain labeled with Gd-BOPTA by improving MRI signal and visualized the distribution of dextran by calculating the diffusion coefficient of the dextran molecule in the brain parenchyma based on fluorescence images. The enhancement of the MRI signal from the blood–brain barrier opening to GD-BOPTA results in diffuse columnar enhancement extending from the top to the bottom of the brain. Focused ultrasound (FUS) is capable of inducing transient local BBB opening effects as a means of brain-targeted drug delivery. Dynamic contrast-enhanced magnetic resonance imaging (DCE-MRI) is the most widely used noninvasive imaging technique to assess the opening/damage of the blood–brain barrier and Chai [57] showed FUS-BBB opening regions with Gd-BOTPA penetrating the CNS with high signal intensity and kinetic parameters K_trans_ (0.0086 at 0.4 MPA pressure min^−1^) to analyze the kinetics of the transport molecules during the opening of FUS-BBB.

MRI has a positional resolution of 50–500 μm, allowing for the location of more precise areas of brain tissue. Using MRI and histology to monitor the location and progression of glioblastoma, cerebral microdialysis probes can be implanted at predetermined reference points to collect microdialysis samples of tumor extracellular fluid and determine redox concentrations to characterize adriamycin concentrations. When ultrasound treatment resulted in the opening of the blood–brain barrier, adriamycin drug uptake was significantly increased in brain tumors, with a three-fold increase in AUC compared to the adriamycin alone group and prolonged residence time of redox in tumor cells [58].

The contribution of MRI to anatomy is outstanding and its ability to provide rapid localization of rat brain tissue structures provides a powerful tool for the field of neuroscience [59]. In terms of observation of pathological features, MRI techniques have become part of the routine diagnostic process to differentiate Parkinson’s disease from secondary or atypical Parkinson’s syndrome due to the high specificity of some MRI features for atypical Parkinson’s syndrome. Novel MRI has been developed with potential as a biomarker for early or even prodromal Parkinson’s disease [60], including neuromelanin imaging (NMI) for analysis of nigrostriatal area, contrast ratio and volume [61] and quantitative susceptibility mapping (QSM), for quantification of iron deposition in the substantia nigra [62]. Dementia with Lewy bodies (DLB) and Alzheimer’s disease overlap in clinical features and this distinction is often challenging. Recent MRI studies have shown that DLB is significantly more impaired in white matter than gray matter and both are heavily involved in Alzheimer’s disease [63]. Such results may be of great benefit in improving the diagnostic accuracy of DLB.

### 4.3. Combined Application of Nuclear Imaging Techniques

Each nuclear imaging technique has its strengths and weaknesses. PET and SPECT favor the tracing of radioactive molecules but acquire images with poor spatial resolution, making it difficult to accurately identify the uptake area. The ability of PET to image without the use of a gamma collimator or aperture gives PET a higher sensitivity compared to that of SPECT instruments. CT and MRI are able to provide more comprehensive three-dimensional spatial information, with MRI’s ability to distinguish soft tissue giving it an outstanding advantage in intracerebral imaging. Compared with CT ionizing radiation, MRI uses magnetic field changes as a means to better avoid damage to cellular structures from ionizing radiation [64]. To examine the uptake of intracerebral drugs more comprehensively and to obtain a higher spatial and temporal resolution, investigators can try to use a combination of mutually complementary imaging techniques.

PET provides limited anatomical information and is controlled by the specific distribution of radionuclides, so it is often necessary to combine it with other techniques that can provide anatomical localization to observe local tissue distribution. PET/CT is a technique that combines functional and structural imaging. Lesniak [65] used radionuclide ^89^Zr-labeled bevacizumab desferrioxamine (BVDFO) by PET/CT dynamic scans and obtained axial, sagittal and coronal positron tomography images, from which it was observed that radioactivity accumulated mainly in the arterial ipsilateral striatum, hippocampus and amygdala after arterial injection of monoclonal antibodies following 25% mannitol perfusion to open the blood–brain barrier. The combined imaging provided a reference for the distribution of the drug in fine structures. As the resolution of fused images increases, the structures that researchers can observe become more minute and detailed. Kang et al. [66] used a high-resolution research tomography-positron emission tomography (HRRT-PET) and 7 T-magnetic resonance imaging fusion system to assess the distribution of ^18^F-fluoro-2-deoxy-D-glucose (FDG) uptake in 51 intracranial structures of interest in canine brain structures. In addition to the analysis of larger regions, such as the midbrain, pons and medulla, relative quantification of uptake was also performed for the refinement of the pons caudalis and longitudinal fibers and the clearly identifiable distribution of fine structural drugs in HRRT-PET and 7T-MRI fusion imaging may become a good tool for the assessment of intracranial disease in dogs.

SPECT, with detectable photon emission from an energetic radionuclide, combined with the three-dimensional spatial information obtained by CT and a radiotracer, can be used to assess drug distribution in larger brain tissues, assessing the good affinity and specificity of brain tissue Aβ plaques of radioactive accumulation [^99m^Tc]15 in the rhesus monkey brain. After crossing the blood–brain barrier, [^99m^Tc]15 brain uptake peaked at 0–10 or 10–20 min after injection, followed by a slow decline with reasonable clearance, which emphasizes the utility of the ^99m^Tc-labeled Aβ probe [67]. In addition to this, the combination of systemic and local SPECT/CT for intrathecal (IT) delivery of antisense oligonucleotides (ASOs) was used to achieve infiltration visualization of the neuraxis. Signal entry into the skull was observed immediately after administration and radioactive signal began to accumulate in the head and neck lymph nodes four hours later. Within seven days, there was high signal retention in the meninges, skull base, cerebellar curtain and superior sagittal sinus regions covering the spinal cord and nerve roots [68].

## 5. Mass Spectrometry Imaging for Assessing Intracerebral Pharmacokinetics

In 2011, the concept of “in situ metabolomics” emphasized the role and mechanisms of metabolites in life activities and pathological processes by characterizing their spatial and temporal distribution [69]. Mass spectrometry imaging (MSI) is a label-free molecular imaging technique that allows for the visualization of any specified mass-to-charge ratio ion (*m*/*z*) in the sample to be measured, to provide information on the spatial localization of drugs and other molecules in biological samples simultaneously. It plays a key role in cerebral pharmacokinetic studies by compensating for the lack of specificity and the cumbersome radiolabeling process of radioactive imaging techniques.

### 5.1. Analysis of Single-Compound Mass Spectrometry Imaging

According to some of the methods that have been created, MSI can be used to provide spatially resolved molecular information at different levels [70]. MSI qualitatively evaluates the drug’s ability to cross the blood–brain barrier and its distribution in brain regions by generating ionic intensity maps of brain tissue samples. MALDI-MSI showed that elvitegravir successfully penetrated BBB and was distributed to almost all regions of the brain. However, relatively high *m*/*z* 448.21 ions were observed in the thalamus, hypothalamus and corpus callosum 1 h after injection [71]. MSI images at various time points enable us to infer the spatial and temporal distribution characteristics of the drug in the brain. Shobo [72] judged from the distribution images within 2 h after Pretomanid administration that the drug can diffuse into the brain through the tight junction of the endothelium of the cerebral capillary network and diffuse from the cortex to the corpus callosum for widespread distribution to other areas of the brain. The concentration of drug in the corpus callosum steadily decreased over 4–8 h, providing evidence of outward diffusion into adjacent areas of the brain.

**Figure 3:**
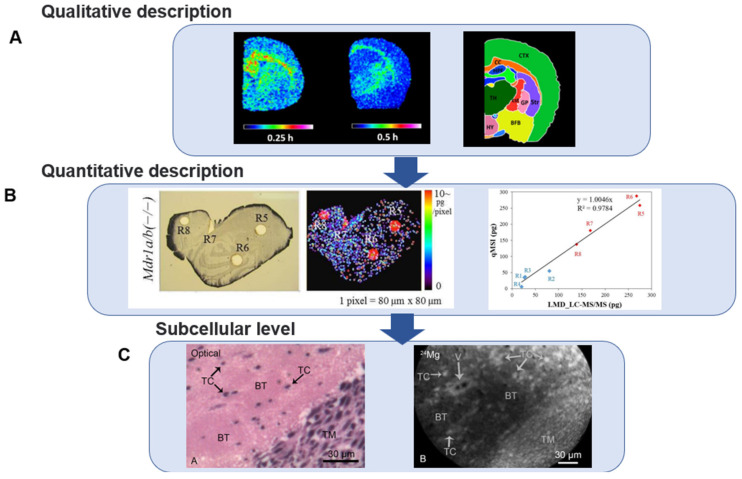
Development process of mass spectrometry imaging technology. (**A**) Qualitative description: Ion images combined with anatomical mapping showed that RPV was able to reach almost all brain regions after administration. A high degree of *m*/*z* 367.0 localization was observed in hippocampal structures (HPF) and corpus callosum (CC) at 0.25 h. Reprinted with permission from Ref. [74]. © 2019, Sphamandla Ntshangase. (**B**) Quantitative description: calibration curves of alectinib standards and images of alectinib distribution in sections of mouse brain. In the calibration curve on the right, R1-R4 (blue) and R5-R8 (red) present FVB (section not shown) and Mdr1a/b KO (section shown on the left) of mouse with different genes, respectively. Reprinted with permission from Ref. [75]. © 2016, HiroakiAikawa. (**C**) Subcellular level: H&E images and SIMS images of magnesium distribution in the brains of F98 glioma rats. H&E image shows the interface between the tumor mass (TM) in brain tissue (BT)of infiltrating tumor cells (TC). SIMS image shows higher magnesium concentrations in TM and TC. Reprinted with permission from Ref. [81]. © 2015, Subhash Chandra.

The region-specific distribution characteristics of the brain are important for predicting drug efficacy. Mdanda [73] investigated the penetration capacity of BBB and brain location of the antiretroviral drug nevirapine (NVP) at the spatial level using MADLI-TOF. Coronal brain tissue sections normalized at tic showed that nvp was able to penetrate the blood–brain barrier at 0.25 h in a wide distribution in the brain, including the cortex, thalamus, hippocampus, cortical pathways, corpus callosum and associated subcortical white matter. After 0.5 h, NVP was strongly expressed only in the cortical pathways and corpus callosum. MALDI-MSI showed that rilpivirine (RPV) was highly enriched in corpus callosum and hippocampal structures and after 2 h MALDI-MSI was unable to detect the drug, as detected by LC-MS/MS [74] (Figure 3A). Both anti-CNS HIV drugs were concentrated in brain regions associated with the development of neurodegenerative lesions, suggesting a longer drug residence time in brain regions to provide a higher degree of CNS efficacy.

MSI evolved from qualitative and semi-quantitative to quantitative imaging and its quantification requires the addition of standard solutions dropwise to sagittal brain tissue sections to obtain standard curves, the evaluation of background signals that may interfere with analytes, correction of local ionization bias of endogenous analytes in different brain structures and the conversion of peak drug intensities to drug concentrations in the corresponding regions in tissue sections. It can be applied to the effect of multidrug-resistance protein 1 (MDR1) on the intracerebral delivery of the drug. At 4 h after 4 mg/kg and 20 mg/kg administration, aletinib showed increased levels of drug accumulation in the brains of knockout mice, which were 20- and 14-times higher than wild-type, respectively [75] (Figure 3B). The maximum spatial resolution of MSI can range from 50 nm to 200–300 μm depending on the ion source and sample characteristics [70]. The high spatial resolution makes it possible to quantitatively analyze MSI in specific regions of brain tissue. Tanaka et al. [76] observed that the concentration of Epertinib in breast cancer IVM brain metastases was more than 10-fold higher than lapatinib after 4 h of administration and the ratio of Epertinib concentration in tumor to normal brain tissue was 4-fold higher than lapatinib.

The heterogeneity in the tumor microenvironment directly affects drug penetration, decreases therapeutic efficacy and promotes the development of drug resistance. Therefore, evaluating drug concentration and distribution is of great importance for the brain and tumors. MSI is able to correlate the drug distribution characteristics observed in solid tumors with tumor heterogeneity [77]. Pokorny [78] analyzed the distribution of MK-1775 in situ and ectopic GBM using MALDI-MSI. The accumulation of MK-1775 in in situ transplanted tumors was significantly lower compared to ectopic tumors and the level of exposure to MK-1775 in some regions was even similar to that of normal brain tissue, suggesting that heterogeneous aggregation of MK-1775 within brain tumors may limit the efficacy of MK-1775 for GBM.

### 5.2. Co-Localization Imaging with Endogenous Molecules

MSI has the specificity characteristics of mass spectrometry and enables high-coverage detection of multiple molecules in organisms by selecting different mass-to-charge ratios. Co-localization of drug distribution signals with endogenous analytes serves as a biomarker for disease progression, therapeutic efficacy or toxicology. Rzagalinski et al. [79] used FTICR mass spectrometry imaging to assess the ability of teriflunomide (TRF) to cross the blood–brain barrier while visualizing potential changes in the spatial and quantitative distribution of endogenous compounds in the CNS under drug action. Differences in purine and pyrimidine nucleotides, as well as glutathione and carbohydrate metabolic intermediates, revealed the potential effects of teriflunomide on metabolic networks in the mouse brain, providing additional insight into the role of the drug at the molecular level.

In addition to this, co-localization of the drug signal with heme (*m*/*z* 616.2) derived from erythrocytes enables further evaluation of drug permeability in the cerebral vasculature [78], providing the ability to accurately assess drug pharmacokinetics in target tissues or organs at the microscopic level. MSI with a resolution of 20 μm examined the position of aletinib in relation to heme and the distribution of aletinib in wild-type mice was mainly co-localized with heme, while a diffuse distribution of aletinib was observed in the brains of mdr1-deficient mice [75], which serves as a useful complement to MSI imaging of general brain tissue.

### 5.3. Microscopic Imaging

Mass spectrometry has significantly advanced in subcellular resolution metabolic imaging, where MSI at the cellular/subcellular level can provide critical details of drug delivery mechanisms, kinetics and high resolution compared to tissue mass spectrometry imaging, allowing a shift from identifying drugs that interact with specific organs to identifying drugs that interact with specific cell types. Recent technological advances in the field of MALDI have increased the resolution to a few micrometers, thus, allowing for the visualization of larger cells and organelles [80]. Chandra [81] used high-resolution (500 nm) secondary ion mass spectrometry (SIMS) elemental/isotope imaging to quantify tumor cells in normal brain tissue adjacent to F98 rat gliomas. The altered magnesium homeostasis of the basement membrane cells was evaluated by approximately doubling the magnesium concentration in the infiltrating tumor cells in the brain compared to the surrounding normal brain tissue (Figure 3C). Analysis of infiltrating tumor cells measured at in situ single-cell resolution could greatly enhance the pathophysiology of GBM. Thus, 3D OrbiSIMS enables molecular imaging of the hippocampal region of the mouse brain on tissue, cellular and subcellular scales for analysis of fatty acids, sterols, glycerophospholipids and sphingolipids, molecules that play important roles in neurodegenerative diseases, superimposed on HE-stained tissue sections to provide complementary molecular pathology [82].

## 6. Conclusions and Future Direction

In this review, we discuss the technical means of imaging in the field of cerebral pharmacokinetics, presenting examples of their technical approaches and applications. To summarize, the detection of compound molecules today starts with their own features or additional features, which are tracked by optical imaging systems if autofluorescence is present. In the absence of distinctive features, the distribution of compounds can also be visualized with a fluorescent or radiolabel attached, depending on the need and cost. The application of imaging techniques is straightforward for the display of molecules across the BBB, avoiding false-positive results from brain homogenization methods and the impact of invasive methods on the integrity of the BBB. Optical imaging systems continue to develop and progress in the exploration of detection bands and are widely used with the advantage of small device sizes. Trapping fluorescent molecules with the brain to determine the physiological and pathological state of the body has become its main application and it also plays an important role in clinical brain tumor resection surgery. Radiographic autoradiography has important implications in the construction of systemic and/or specific tissue kinetic models. The focus on drug accumulation in target tissues can also take into account the entry of non-target drug molecules, helping to assess toxicology, pharmacology and drug disposition. MARG has improved resolution on the basis of WAB and is able to focus on finer structures and provide a more comprehensive interpretation of the distribution of drugs in brain tissue and brain tumors. Nuclear imaging techniques are capable of detecting isotopically labeled molecules in the brain at the in vivo level, and multiple nuclear imaging techniques combine and complement each other depending on their function to provide access to three-dimensional spatial molecular information for brain tissue. Due to the tediousness of the labeling process, the selection and imaging of molecules with their own mass-to-charge ratio appears simpler and more straightforward. The specific mass-to-charge ratio selection feature of MSI greatly enhances the specificity of imaging and, in addition to distinguishing drug molecules from metabolites, co-localization with endogenous molecules makes it convenient for further studies of pharmacodynamics (Table 1).

In recent years, the concept of cellular pharmacokinetics [83] has been proposed due to the fact that most drug-metabolizing enzymes and drug targets are located inside cells [84]. Professor K.L.R Brouwer, an internationally renowned expert in pharmacokinetics, also gave a high evaluation [85]. Optical imaging, radiography and mass spectrometry imaging techniques have also become powerful tools for monitoring molecules at the cellular/subcellular level in brain tissue in vitro, promoting in vitro to in vivo prediction, translation and resolution of possible PD/PK inconsistencies in tissue pharmacokinetics. The combined application of the above technologies in vivo and in vitro will make the pharmacokinetic study in the brain more comprehensive and complete and will gradually form a comprehensive “subcellular-cellular-tissue” pharmacokinetic study system in the brain, which can guide the clinical application of brain-targeted drugs. At the same time, it can also prevent the toxicity and damage of non-brain-targeted drugs to brain tissues and become one of the important technical systems to support future drug development.

## Figures and Tables

**Table 1:** Comparison of imaging techniques for the assessment of cerebral pharmacokinetics.

Analysis Method		State of the Sample	Spatial Resolution	Cost	Safety	Application
Optical Imaging	Microscopy	Isolated tissue	10–200 μm	Low	Good	Visualization of drugs in whole or finer structured brain tissue
	NIR-I	Living	1–2 mm	Medium	Good	Overall/local brain tissue visualization
	NIR-II	Living	Range from 10 μm to 1–2 mm	Medium	Good	Visualization of cerebrovascular and intracerebral tumors
Autoradiography	WBA	Frozen section	50–100 μm	High (time)	Medium	Overall/local brain tissue visualization
	MARG	Frozen section	Can reach 1 μm	High (time)	Medium	Cellular/receptor localization
Radionuclide imaging	PET	Living	1–2 mm	High	Medium	Joint application: overall/local brain tissue visualization
	MRI	Living	50–500 μm	High	Medium	
	CT	Living	10–500 μm	Medium	Medium	
MSI		Frozen section	Range from 50 nm to 200–300 μm	High	Good	Single/multiple molecules co-localization visualization
